# A Critical Review of the Decarbonisation Potential in the U.K. Cement Industry

**DOI:** 10.3390/ma18020292

**Published:** 2025-01-10

**Authors:** Ziyad Sherif, Shoaib Sarfraz, Mark Jolly, Konstantinos Salonitis

**Affiliations:** Sustainable Manufacturing Systems Centre, Faculty of Engineering and Applied Sciences, Cranfield University, Cranfield MK43 0AL, UK; z.sherif@cranfield.ac.uk (Z.S.); s.sarfraz@bham.ac.uk (S.S.); m.r.jolly@cranfield.ac.uk (M.J.)

**Keywords:** green cement, cementitious materials, CO_2_ emissions, energy, decarbonisation, U.K. cement industry

## Abstract

As urbanisation and infrastructure development continue to drive rising cement demand, the imperative to significantly reduce emissions from this emissions-intensive sector has become increasingly urgent, especially in the context of global climate goals such as achieving net zero emissions by 2050. This review examines the status, challenges and prospects of low-carbon cement technologies and mitigation strategies through the lens of the U.K. cement industry. A mixed-methods approach was employed, combining structured literature searches across academic databases with analyses of industry reports, market data and technological roadmaps to ensure a comprehensive evaluation. Following an outline of cement production, resource flows and the sector’s landscape in the U.K., the review delves into an array of decarbonisation pathways. This includes deploying the best available technologies (BATs), fuel switching, carbon capture utilisation and storage (CCUS), clinker substitution and low-carbon cement formulations. A critical assessment is provided on the technological readiness, costs, resource availability considerations and scalability aspects governing the widespread implementation prospects of these approaches within the U.K. cement industry. Furthermore, this study proposes a roadmap that considers priority avenues and policy needs essential for facilitating the transition towards sustainable cement production aligned with the U.K.’s net zero obligations by 2050. This evaluation contributes significantly to the ongoing decarbonisation discourse by holistically mapping technological solutions and strategic imperatives tailored to the unique challenges and opportunities presented by the U.K. cement sector.

## 1. Introduction

Cement production is a globally crucial yet highly emissions-intensive sector. Concrete, with cement as its primary constituent, is the second most consumed material worldwide after water due to its extensive use in construction [1,2]. Over the past two decades, global cement demand has surged four-fold, exceeding 4 billion tonnes annually [3]. This rapid growth trajectory has positioned the cement industry as a significant contributor to global greenhouse gas (GHG) emissions, accounting for 7% to 9% of anthropogenic carbon dioxide (CO_2_) emissions [4,5,6]. Moreover, the cement sector is the second-largest industrial CO_2_ emitter after the iron and steel sector. With global urbanisation and development trends set to increase cement demand further, the cement industry is projected to witness a significant 4% surge in its direct CO_2_ emissions on a global scale by the year 2050 [7]. This presents a severe sustainability challenge and material constraint on future development pathways in the context of climate change mitigation efforts.

**Figure 1:**
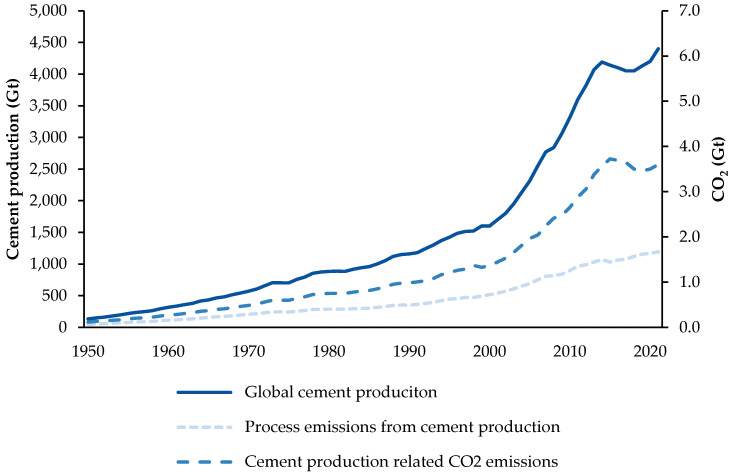
Cement production and CO_2_ emissions trends. Data compiled from [4,10,11].

Global cement production and the associated CO_2_ emissions have steadily grown over the past few decades, driven by rising populations, accelerating urbanisation rates and increasing infrastructure development needs worldwide, except for temporary declines during eras of economic crisis (Figure 1). Projections indicate that global cement demand will increase by 12–23% by 2050 compared to 2020 [8]. Similarly, in the United Kingdom (U.K.), a 4.6% growth in cement demand is expected by 2030 [9]. Given the anticipated growth trajectory and the global agenda to achieve net zero emissions by 2050, there is a critical imperative to significantly reduce CO_2_ emissions from cement manufacturing by 24% by mid-century [7].

**Figure 2:**
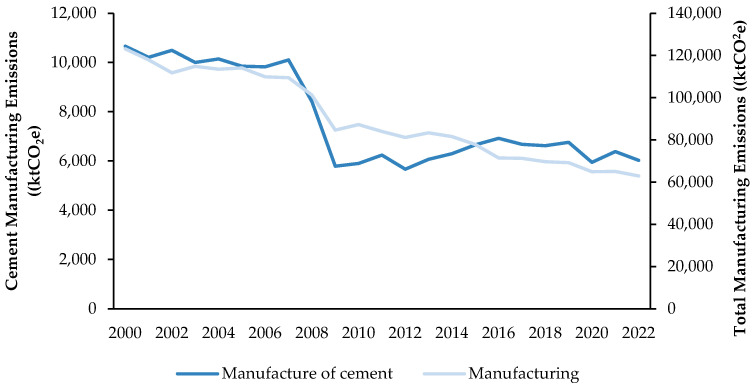
The emissions of U.K. manufacturing against cement manufacturing. Data collected from [17].

Cement manufacturing in the U.K. has remained relatively stable since an initial decline following the 2008 economic crash, unlike the broader manufacturing sector, which has experienced a declining trend over the past two decades [12,13]. The cement industry produces around 9 million tonnes of cement annually [14], emitting approximately 7.3 million tonnes of CO_2_ annually [15]. Unlike the broader manufacturing sector, the cement industry has demonstrated a lower rate of decline in emissions. Despite reducing emissions by 53% since 1990 and decarbonising at a faster rate than the U.K. economy overall [16], emissions have trended upward since 2009. This increase is driven by heightened industry stability and growing construction demands, leading to a rise in cement production (Figure 2).

**Figure 3:**
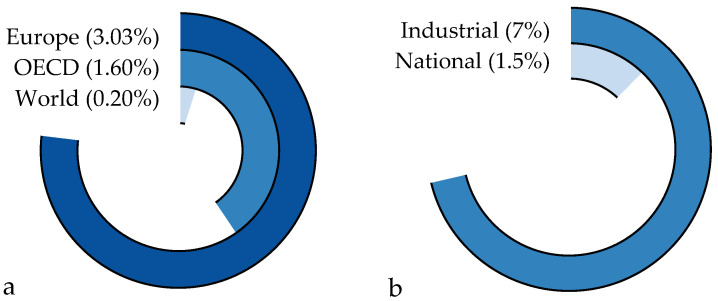
U.K.’s overview. (**a**) U.K.’s production as a portion of the broader regions and (**b**) U.K.’s cement sector emissions as a portion of broader aspects. Data extracted from [21,22,23,24].

Given the U.K.’s high self-reliance on its manufacturing capabilities, producing over 70% of its cement domestically and importing the remainder [18], the cement production emissions, though relatively small compared to broader regions (Figure 3a), constitute a significant portion of the country’s total emissions. Specifically, these emissions account for approximately 1.5% of the U.K.’s national and 7% of its industrial carbon emissions (Figure 3b). 

Under the 2008 Climate Change Act stipulations and subsequent carbon budgets, the U.K. is legally obligated to achieve net zero territorial carbon emissions by 2050 [19,20]. Interim targets include a 78% reduction in emissions against 1990 levels by 2035 across the whole economy. Reaching these strict decarbonisation goals across the heavily industrialised U.K. economy will necessitate radical emissions cuts in hard-to-abate, carbon-intensive sectors like cement manufacturing. 

To chart a path towards deep decarbonisation, the U.K. cement and concrete industries have published various technology roadmaps and decarbonisation strategies in recent years [15,25,26]. These roadmaps heavily rely on two principal technological approaches—deploying carbon capture, utilisation and storage (CCUS) systems and reducing clinker content in cement by promoting widespread substitution with cement constituents like fly ash and ground granulated blast furnace slag (GGBS). While these materials are commonly referred to as supplementary cementitious materials (SCMs) in the broader literature, they are classified as cement constituents under BS EN 197-1:2011 when used as main components in blended cement [27].

However, both core strategies face considerable barriers and limitations regarding costs, technology readiness, resource availability constraints and uncertainties regarding their potential for achieving the absolute emissions reductions required to align with net zero obligations [28,29].

While decarbonisation technologies and strategies have been explored globally, there remains a lack of a focused, systematic review that addresses the specific challenges, opportunities and technological pathways for decarbonising the U.K. cement industry. Most existing studies provide fragmented perspectives, often neglecting the contextual nuances of the U.K.’s emissions profile, manufacturing practices and policy landscape. This critical review bridges that gap by evaluating low-carbon cement technologies and mitigation strategies through the specific lens of the U.K. cement industry.

Several other levers for emissions mitigation in cement production also exist but have limitations regarding absolute reduction potentials. These include improving energy efficiency across manufacturing processes, integrating renewable energy sources like solar and wind power, employing alternative fuels like biomass and waste-derived inputs and adopting state-of-the-art best available technologies (BATs) [30]. Consequently, transformative low-carbon “green” cement formulations and alternative binder technologies are attracting significant research interest and investment as potential long-term solutions to facilitate deeper decarbonisation of the sector [31].

This critical review addresses the existing gap in the literature by examining the status, research frontiers and prospects of low-carbon cement technologies and mitigation strategies specific to the U.K. cement industry. While global studies exist, focused insights into the U.K.’s unique emissions profile, manufacturing practices and resources landscape remain limited. This review highlights the challenges, opportunities and technological pathways required to meet the U.K.’s emissions reduction targets. Moreover, abatement options and recommendations are identified, along with policy needs necessary to facilitate widespread future implementation of these low-carbon pathways across the U.K. cement industry.

## 2. Methodology

This review examines the decarbonisation potential within the U.K. cement industry through a mixed-methods approach, addressing the lack of extensive academic literature specific to this context. The methodology adopted here diverges from traditional systematic review frameworks such as PRISMA due to the limited availability of relevant academic studies. Instead, a hybrid approach was used, combining academic literature searches, industry reports and market data analyses. The steps undertaken to ensure a rigorous and comprehensive review process are detailed below.

### 2.1. Data Sources and Selection Process

Academic Literature Search: Searches were conducted in Scopus and Web of Science, using broad terms to capture relevant papers on decarbonisation in the cement industry globally. Keywords included combinations of “decarbonisation”, “cement” and “UK”. The search yielded a total of 112 papers, of which 17 were deemed relevant after screening.

Industry Reports and Trade Association Publications: Recognising the scarcity of U.K.-specific academic studies, reports from industry bodies such as the Mineral Products Association (MPA), the Global Cement and Concrete Association (GCCA) and government agencies were incorporated. These sources provided critical insights into current practices, technological developments and policy frameworks specific to the U.K.

Speciality Market Data: Data on production capacities, emission profiles and market segmentation of U.K. cement manufacturers were accessed through IBISWorld, Statista and proprietary databases. These datasets were cross-referenced with academic sources to ensure consistency and reliability.

### 2.2. Inclusion Criteria

The studies or reports were included if they covered applicability to cement production technologies and directly addressed decarbonisation strategies, including fuel switching, carbon capture and clinker substitution. Moreover, sources had to be specific to the U.K. cement sector or contain transferable insights applicable to the region. Finally, publications should have been made within the last 10 years, supplemented by older studies foundational to technological or procedure developments.

### 2.3. Review and Analysis Process

#### 2.3.1. Thematic Progression

**Figure 4:**
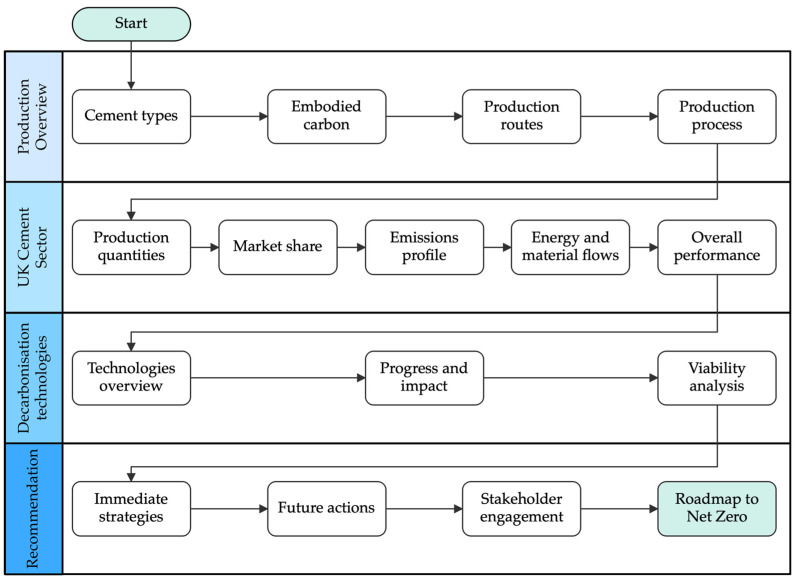
Overview of the key steps and thematic areas covered in this review.

To ensure a clear and cohesive narrative, the review focused on analysing the interconnected challenges and opportunities within the U.K. cement industry. These were organised into three overarching themes: cement production attributes, technological innovations and future considerations. An outline highlighting the sequential steps and thematic focus areas of decarbonisation strategies in the U.K. cement industry is shown in Figure 4.

The review commenced by contextualising the industry’s production dynamics through mapping cement varieties and manufacturing processes. Afterwards, a characterisation of the U.K.’s cement sector took place; this included the examination of market performance and emissions profile, emphasising the material and energy flows unique to the sector. This foundational understanding sets the stage for exploring decarbonisation strategies in depth. The investigation transitions from broad contextual analysis to specific approaches, which were categorised into key decarbonisation strategies: (1) BATs, (2) fuel switching, (3) CCUS, (4) clinker substitution and (5) emerging innovations. Each pathway was assessed for technological readiness, environmental impact, cost and validity aspects that govern its large-scale deployment prospects within the U.K. cement sector. This approach ensured that the findings were accessible and actionable for both academic and industry stakeholders. Lastly, the findings were used to build the bases for a decarbonisation roadmap specific to the cement sector in the U.K., framed by the interplay of technological viability and stakeholder engagement.

#### 2.3.2. Quantitative and Qualitative Data Synthesis

A combination of quantitative metrics (e.g., emission reductions, cost per tonne of CO_2_ mitigated) and qualitative evaluations (e.g., policy and operational barriers) was used. Key findings were summarised in visual tools. For instance, Sankey diagrams were utilised to portray the energy and material flows, while a four-quadrant cost–impact matrix and a star rating table were produced to emphasise the intricate factors concerning decarbonisation technologies. These tools were developed by compiling data from multiple sources and normalising them to ensure comparability.

### 2.4. Limitations

This methodology acknowledges potential limitations, including the following:

- Limited availability of peer-reviewed studies focused exclusively on the U.K. cement industry;
- Variability in the accuracy of industrial and market reports;
- Challenges in applying global insights to the specific U.K. context.

These limitations were mitigated by prioritising robust, cross-validated sources and transparently reporting assumptions where applicable. By combining academic and non-academic sources, this mixed-methods approach provides a comprehensive and nuanced perspective on decarbonisation in the U.K. cement sector, tailored to address the unique challenges and opportunities within this industry.

## 3. Cement Overview

Cement plays a pivotal role in the construction industry, comprising 15% of concrete’s composition, making it one of the most widely used materials in the world [5,30]. Despite its minor percentage, cement has by far the highest carbon footprint per unit mass compared to any other concrete constituent [32]. According to the European Standard EN 197-1, which defines and provides specifications for cement types, there are five families of cement products, further divided into 27 common types of cement [27].

**Figure 5:**
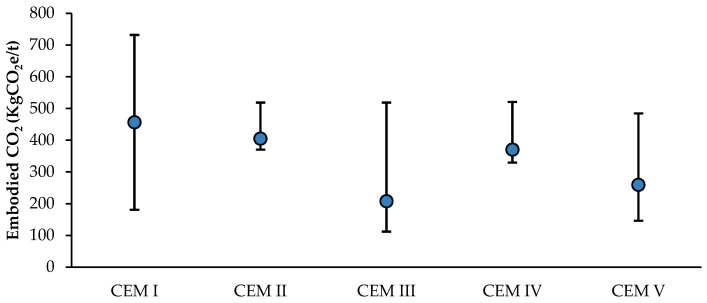
Cradle-to-gate embodied carbon by cement types showing average values (dots) and the range of minimum to maximum values (whiskers). Data extracted from [25].

The definitive difference between the types is the varying quantities of clinker content and accompanying cement constituents. Figure 5 presents the five cement families’ average, maximum and minimum embodied carbon. CEM I, commonly called Portland cement, primarily consisting of 95% clinker and 5% gypsum, stands out for its high carbon intensity. Additionally, it is also the most commonly used type of cement in the U.K. [33]. However, the wide range of embodied carbon values across these types introduces considerable uncertainty, underscoring the need for more precise data. Adopting digital product passports could help standardise and clarify the environmental impact of these materials, providing more consistent and reliable information for stakeholders [34,35].

**Figure 6:**
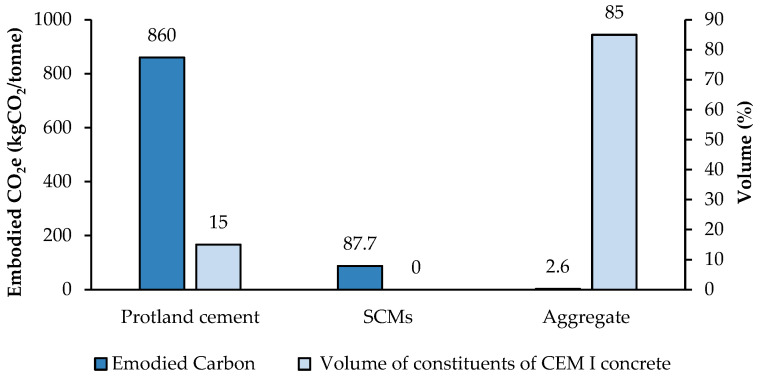
Embodied CO_2_e emissions from main concrete constituents and CEM I concrete volume. Data extracted from [37].

Concrete manufacturing involves blending cement with water and aggregates. Figure 6 presents the embodied emissions of typical constituents of concrete, as well as the volume of constituents in a CEM I concrete. It is evident that the portion of Portland cement contributes significantly to emissions despite its fractional use. SCMs, which act as substitutions for cement in other, less utilised concretes, embody approximately one-tenth of the carbon emissions. Therefore, Portland cement substitution by SCMs can significantly reduce CO_2_ intensity from cement production even by 75% [36].

### 3.1. Cement Production Methods

In cement production, four principal techniques are used to process raw materials: dry, semi-dry, semi-wet and wet. Each method is tailored to accommodate specific raw material characteristics and embodies varying energy requirements.

The dry method involves grinding raw materials without water and feeding the finished product into a rotary kiln. In the semi-dry method, dry raw materials are pelletised with a small amount of water, pre-heated and calcined before ground into powder. This method is preferred for raw materials with high alkali concentrations. Alternatively, the semi-wet method uses wet cakes of raw materials, which are filtered to remove excess water and ground into powder after drying. This approach is suitable when raw materials have moderate plasticity. Finally, the wet method is employed for raw materials with high moisture content, where a wet slurry is introduced into a rotary kiln and dried using a separate system, requiring significant fuel but less electrical energy [38].

**Figure 7:**
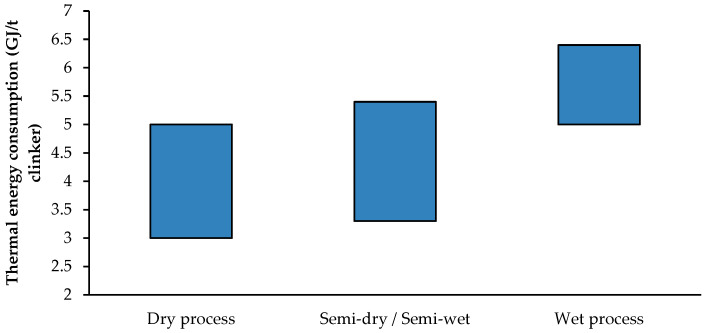
Fuel energy use in cement manufacturing. Data extracted from [38].

Figure 7 presents the range of energy consumption for each method. The most common approach, as the literature indicates, is the dry method. This method is preferred due to its lower carbon dioxide emissions, reduced fuel consumption, lower production costs and shorter processing times [39]. Despite its prevalence, resource constraints and environmental sustainability challenges persist, prompting research into more sustainable practices and technologies [40,41,42]. However, the lack of precise data on energy consumption and emissions introduces uncertainty in calculations, particularly for life cycle assessments (LCAs). This uncertainty can impact the accuracy of environmental impact evaluations, potentially underestimating or overestimating the benefits of specific methods. More robust and reliable data collection is essential to ensure that LCA results reflect the environmental implications of different production methods, thereby guiding better-informed decisions for sustainable practices.

### 3.2. Cement Production Process

**Figure 8:**
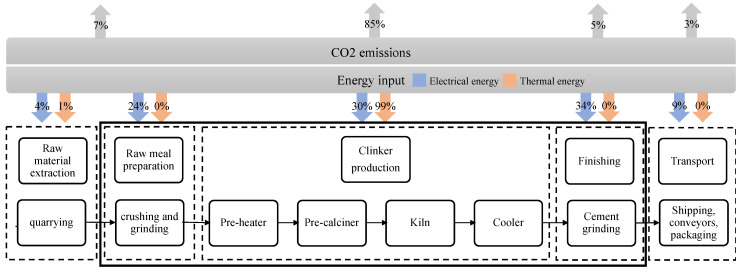
The typical cement production process along with energy (electrical and thermal) and carbon emissions distribution. Data extracted from [46,47].

The cement production process involves several stages, starting with raw material extraction, grinding, sintering and clinker formation, followed by grinding into cement. Figure 8 illustrates this production process and the distribution of energy consumption (thermal and electrical) and carbon emissions across its various sub-processes. The emissions from cement production arise from different sources. Around 40% comes from burning fossil fuels to generate the thermal energy needed to achieve the high temperatures. Moreover, chemical processes during clinker production release around 50–60% of emissions. Additional energy needs for extraction and transportation contribute around 10% to emissions (7% resulting from raw meal extraction and preparation while 3% from transportation) [43].

Clinker production is at the core of the cement manufacturing process and is the primary source of emissions in cement manufacturing. It is estimated that roughly 0.8 tonnes of CO_2_ are emitted for every tonne of clinker produced [36]. This crucial component is formed during the sintering stage and is characterised by high emissions due to process reactions and substantial energy demand [44]. During this stage, also known as calcination, limestone is subjected to temperatures exceeding 1450 °C, which decomposes calcium carbonate (CaCO_3_) into calcium oxide (CaO) and CO_2_. Calcination contributes substantially to CO_2_ emissions, accounting for approximately 50% of the total emissions generated during cement production [6,45]. Due to the high temperatures, clinker production is also the primary thermal energy consumer (99%). Therefore, fossil fuel combustion is pivotal in emitting CO_2_ during cement manufacturing. They are commonly used as an energy source to heat the cement kiln. The combustion of these fossil fuels releases CO_2_ into the atmosphere, contributing to the 40% of emissions associated with cement production [29]. Moreover, the energy needed for all preceding and subsequent processes, such as raw material extraction, grinding and transportation, also involves the combustion of substantial quantities of fossil fuels, contributing to approximately 10% of CO_2_ emissions [46].

The energy and emissions-intensive nature of cement production presents a significant challenge for the cement industry and the various sectors indirectly reliant on it, particularly the construction industry. Addressing emissions from the two critical sources (calcination during clinker production and fossil fuel combustion) is essential for mitigating the environmental impact of cement manufacturing. Before investigating specific decarbonisation methods and strategies, it is imperative to gain insight into the dynamics of the U.K. cement sector.

### 3.3. U.K. Cement Sector

The U.K. cement and concrete sectors employ 74,000 individuals and contribute a significant GBP 18 billion to the country’s GDP [44]. Moreover, over 95% of concrete is domestically manufactured, underscoring the nation’s heavy reliance on cement production [15].

**Figure 9:**
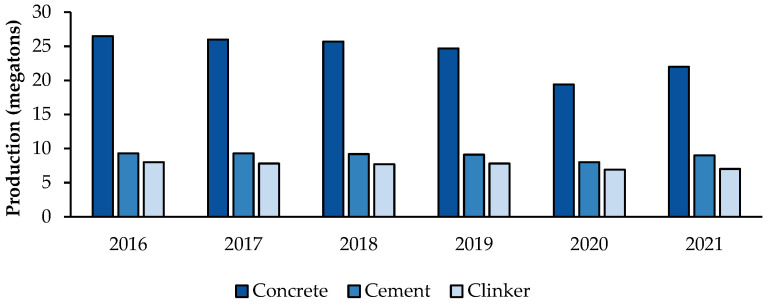
Cement and concrete production in the U.K. Data collected from [48].

As depicted in Figure 9, the U.K. produced 22 million tonnes of concrete, 9 million tonnes of cement and 7 million tonnes of clinker in 2021. While the total values have fluctuated, the ratios have stayed relatively consistent. This highlights the substantial reliance on clinker-based cement.

**Figure 10:**
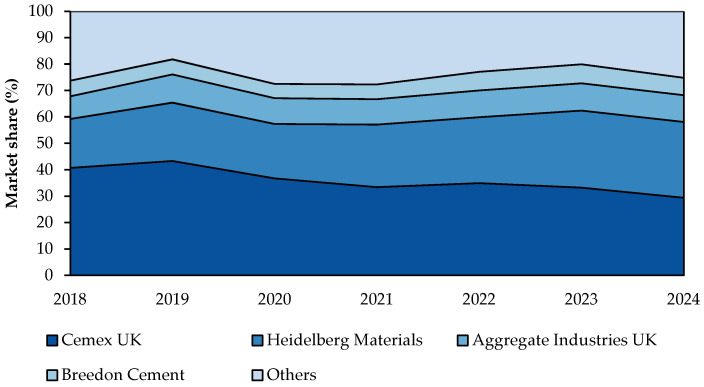
Market share of U.K. cement manufacturers. Data extracted from [9].

In fact, CEM I is the primary type of cement produced in the U.K., representing 79% of all cement produced in the market in 2021 [49]. The five largest cement manufacturers in the U.K. are Cemex U.K., Heidelberg Materials U.K., Aggregate Industries U.K. and Breedon Cement, which account for 75% of the market, as presented in Figure 10. They are subsidiaries of multinational parent companies, with their headquarters and research centres located overseas, mainly in Europe. Together, they operate a total of 11 kiln sites nationwide [50,51].

This market share concentration among a few key players indicates the limited number of companies operating in the field. While this may pose challenges for new market entrants, it also suggests that implementing changes within the sector could be more straightforward due to the smaller number of major corporations. Moreover, large manufacturers control subsidiaries across the entire supply chain, from raw materials extraction to distribution networks and construction services [9]. This integrated control further facilitates implementing changes within the sector as a whole.

**Figure 11:**
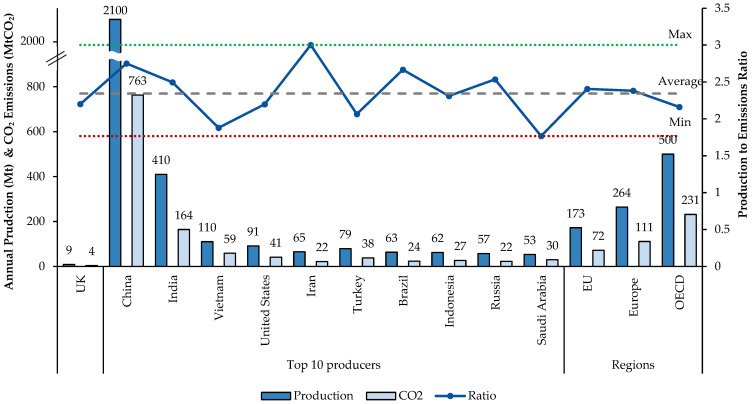
Production and emissions as well as their ratio in 2022 for the U.K., the top 10 cement-producing countries and regions associated with the U.K. A higher ratio equates to better performance. Data compiled from [21,22,52,53,54,55].

To ascertain the U.K.’s position in terms of carbon emissions, the ratio of total production to emissions was compared with that of the top 10 cement-producing countries, as well as regions such as Europe (including EU and U.K.), the OECD and the EU. The results in Figure 11 show that while the U.K. performs better than some major producers, it still falls below the calculated average production-to-emissions ratio.

This highlights the U.K.’s lack of proficiency in this context, indicating that, despite its relatively lower production volumes, the U.K. is not optimising its processes as effectively as other countries with similar or even higher production levels. Inefficiencies in areas such as energy usage, production technology and emissions management strategies are possible causes of the discrepancy. This gap underscores a need for more advanced technologies, stricter policies and innovative practices to reduce emissions per unit of production. By enhancing these areas, the U.K. could potentially lower its emissions footprint and move closer to industry-leading practices, ensuring a more sustainable and competitive position in the global cement industry. Material and energy flows are analysed in the next section to investigate the underlying causes of the substandard circumstance further.

#### 3.3.1. U.K. Cement Energy and Material Flow Analysis

**Figure 12:**
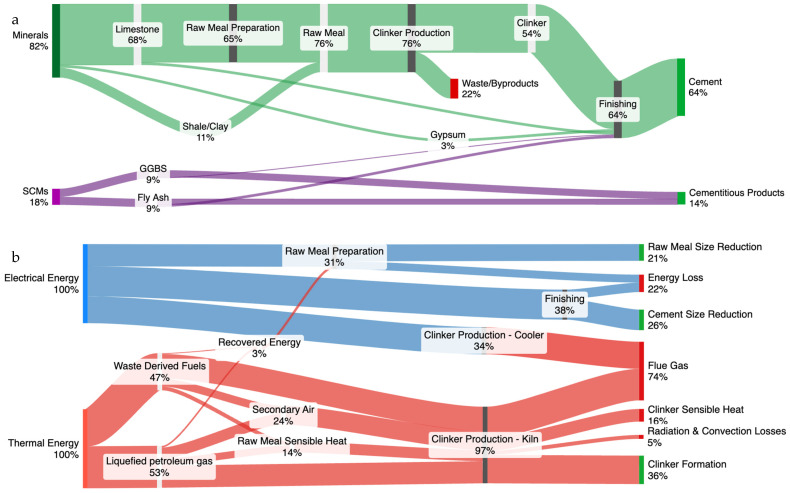
Sankey diagrams of the (**a**) material and (**b**) energy flows. Input values are from sources pertaining to the U.K. cement sector, while the subsequent distribution of flows throughout the processes is computed from average processing data found in the literature. Data compiled as follows: For (**a**), material inputs from [54] and material consumption from [55]. For (**b**), energy inputs from [56] and energy distribution averages from [57,58,59,60,61].

Sankey diagrams were established based on U.K. inputs and average mass and energy balance values to provide a more comprehensive understanding of the energy and material flows in the cement production process. Figure 12a illustrates the material flow in the U.K.’s cement production, emphasising the significant inputs and outputs at each stage. The inputs consist of 82% minerals, primarily limestone and 18% SCMs, which include GGBS and fly ash—both byproducts of other manufacturing processes, such as steel production. Out of the total mineral input, 54% is used to produce clinker, while the remainder is released as waste or byproducts. Of the SCMs, 4% are combined with the clinker to produce cement, while the rest are distributed as cementitious products used in other commodities like ready-mix concrete [56,57]. A substantial portion of inputs are minerals, with limestone being the primary raw material. The integration of SCMs, although currently limited, highlights an area with potential for increasing sustainability by reducing reliance on natural mineral resources and lowering the carbon footprint of the cement industry. Enhancing the proportion of SCMs in cement production could lead to significant reductions in CO_2_ emissions, aligning with the U.K.’s decarbonisation targets.

Similarly, Figure 12b illustrates the energy flow in the cement production process in the U.K., showcasing the different energy sources and their distribution throughout the production stages. Electrical energy is extensively used in raw meal preparation (28%), finishing (38%) and clinker production cooling (34%). Thermal energy, derived from 43% liquefied petroleum gas (LPG) and 57% fuel derived from waste, is predominantly used in the kiln for clinker formation (36%), with the rest being waste heat [58,59,60,61,62,63]. The energy flow diagram indicates heavy reliance on electrical and thermal energy. The prominence of these energy sources in clinker formation underscores the high energy demands and emissions at this stage. While using waste-derived fuels is a positive step towards sustainability, the substantial reliance on coal highlights the need for cleaner energy sources. Enhancing energy efficiency and increasing the share of renewable energy could significantly cut the cement industry’s carbon footprint. In terms of electrical energy demand, it is worth noting that while some companies report purchasing 100% renewable electricity, specific data on the proportion of renewable electricity used across the entire U.K. cement sector are not readily available [64]. This variability in the energy mix means that the actual impact of electricity use on emissions can fluctuate significantly depending on the source of the electricity.

#### 3.3.2. U.K. Concrete Sector Performance

The concrete industry conducts annual performance reporting, which encompasses inputs from various sectors, including aggregate, cement, GGBS, fly ash and admixtures, as well as ready-mixed and precast concrete. These reports cover various indicators such as resource utilisation, waste minimisation and carbon emissions [65]. This initiative commenced with the launch of the Concrete Industry Sustainable Construction Strategy in 2008 and featured targets to be met in 2012. It was then revised with targets set for 2020, and an updated strategy with 2030 targets is currently in development [66].

While the primary focus is on analysing the cement sector, data from the concrete industry were examined due to data availability constraints. Nevertheless, given that cement is a primary component of concrete and is responsible for most emissions, these values offer valuable insights into the sector’s performance. The data encompass the performance of cement, fine and coarse aggregates and omit steel reinforcement.

**Figure 13:**
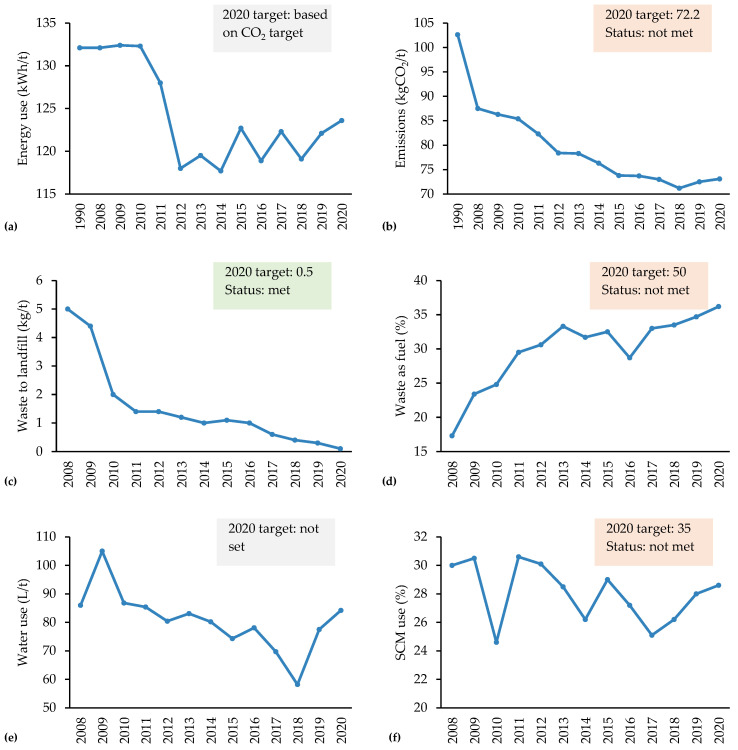
U.K. concrete sector environmental performance for (**a**) energy consumption, (**b**) emissions, (**c**) waste delivered to landfill, (**d**) waste used as fuel, (**e**) water utilisation and (**f**) SCM use. Graphs also display established 2020 targets with green indicating target met, red denotes target not met and grey means that no target was set for this indicator. Data collected from [75].

Figure 13 depicts trends over the past decade for various sustainability indicators, including energy, emissions, waste management, water use and SCM utilisation.

Regarding energy consumption (Figure 13a), values remained relatively consistent from the 1990s to 2010, after which there has been an overall reduction of around 6%. However, this trend has remained somewhat stable. For instance, focusing on data from 2012 onward, energy use shows fluctuations and an upward trend in specific years. These inconsistencies may be influenced by factors such as operational variations, production demands and changes in energy sourcing. The initial reduction after 2010 may be linked to the targets introduced in 2008, which drove industries to lower their energy consumption. However, since emissions reductions are not solely dependent on energy usage, industries may have deprioritised further energy reductions in favour of other emissions reduction strategies.

Carbon emissions (Figure 13b) exhibit a steady downward trend, with emissions per tonne decreasing by 30% over the period. This reduction is likely due to the increased use of renewable energy and low-carbon sources within the concrete industry, along with gradual improvements in production efficiency. However, the 2020 target of 72.2 kg CO_2_ per tonne was not achieved, indicating that further reductions are necessary. The reduction rate has also slowed, likely due to challenges in decarbonising heavy industries like cement, which face technological, economic and policy-related obstacles [67]. High costs and scalability issues hinder the adoption of advanced technologies, such as carbon capture and fossil-free production methods.

Additionally, substantial investments are required for low-carbon transitions, which industries may hesitate to make without assured returns [68]. Policy limitations, including insufficient support for low-carbon innovation and a lack of stable carbon pricing, further slow progress [69]. Despite these barriers, examples like Sweden show that with strong policies and financial incentives, deeper decarbonisation is possible, offering a model for other regions to follow [70].

Waste minimisation has seen significant improvement, particularly in waste management practices. Waste to landfill (Figure 13c) has decreased by 98%, successfully meeting the 2020 target of 0.5%. This dramatic reduction is likely due to policy drivers, such as landfill taxation, which have incentivised the industry to adopt more sustainable waste management practices [71], for instance, the use of waste-derived fuel [72], co-processing clinker and waste in kilns [73] and the use of recycled aggregates [74]. These practices not only divert waste from landfills but also reduce CO_2_ emissions and environmental impacts, with studies showing that co-processing extends landfill life and using recycled aggregates can reduce the demand for natural resources without compromising concrete quality. Congruently, the proportion of waste utilised as fuel (Figure 13d) has concurrently increased by 19% since 2008. Conversely, the target for waste as fuel (50%) was not met. These trends highlight the industry’s efforts towards sustainable waste practices, although with room for further improvement.

Nevertheless, water consumption (Figure 13e) reduction has been relatively modest, with only a 2% decrease observed, a value that may fall within the statistical margin of error. Given the minor reduction and the lack of a defined target for water use, this indicator suggests that water management has not been a major focus area within the industry’s sustainability initiatives. Future strategies could benefit from prioritising water efficiency measures, especially as environmental concerns over water resources grow.

Regarding SCM utilisation (Figure 13f), there has been a relatively consistent utilisation rate, averaging 27%, which fell short of the 2020 target of 35%. The consistent reliance on Portland cement despite available alternatives suggests barriers to wider SCM adoption, potentially due to technical, regulatory or cost considerations. Greater emphasis on diversifying cement types and increasing SCM use could further enhance sustainability within the sector.

## 4. Decarbonisation Technologies and Their Progress

**Figure 14:**
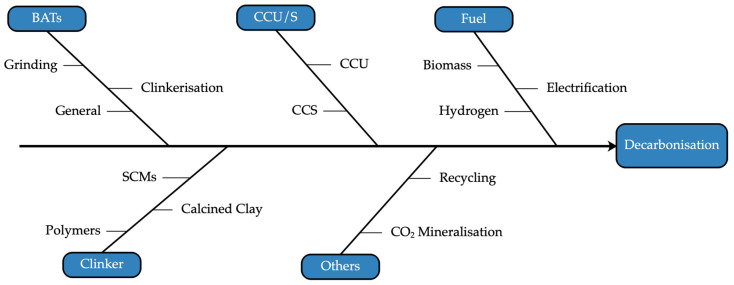
Decarbonisation technologies overview.

The U.K. concrete and cement industry is actively following a roadmap developed in 2020 towards achieving net zero emissions and potentially net negative emissions by 2050 [75]. This involves leveraging various strategies such as decarbonising electricity and transport networks, fuel switching, increasing the use of low-carbon cement and concretes and implementing carbon capture technology in cement manufacturing (Figure 14).

Considerable progress has already been made, with absolute direct and indirect emissions reductions, owing to fuel switching investments, product formulation changes and energy efficiency improvements. Sector-wide initiatives led by the MPA focus on innovation projects aimed at decarbonising cement and concrete manufacturing processes. These projects include exploring alternative fuels, such as hydrogen and plasma technology and developing low-carbon cement with significantly lower embodied CO_2_ emissions. The industry anticipates these advancements will substantially decrease carbon emissions and support the transition towards a more sustainable and environmentally friendly cement sector [15,76].

The following subsections explore the potential and progress of several promising approaches, including adopting BATs, fuel switching, CCUS and clinker substitution. An evaluation of their applicability, effectiveness and challenges within the U.K. context is also provided.

### 4.1. Best Available Technologies

The adoption of BATs in cement production holds significant promise for enhancing energy efficiency, reducing emissions and optimising resource utilisation. BATs encompass diverse technological solutions and operational practices tailored to specific processes and plant configurations.

Regarding energy efficiency, BATs involve implementing advanced process control systems, adopting waste heat recovery systems and optimising kiln operations [38,77]. These measures reduce energy consumption and associated emissions while enhancing overall process efficiency. BATs focus on minimising air pollutants and greenhouse gas emissions to address emissions reduction. Strategies include deploying advanced emissions monitoring and control systems, adopting selective catalytic reduction (SCR) for NOx control and optimising combustion processes [38]. For resource optimisation, BATs aim to minimise the consumption of raw materials, water and energy while promoting alternative fuels and the recovery of valuable byproducts. Approaches include utilising alternative raw materials, recycling process water and harnessing waste heat for power generation [38].

In the U.K., the cement industry has made notable advances in implementing BATs, driven by regulatory requirements and industry initiatives [78]. Noteworthy examples include Cemex and Heidelberg Materials, which have made substantial investments in upgrading equipment within their facilities [79,80]. Specifically, enhancements to grinding mills and blending facilities have been implemented to improve efficiency, increase clean energy utilisation (100%) and reduce emissions (32% reduction). Nevertheless, the widespread adoption of cutting-edge technologies across the sectors faces challenges, particularly concerning high capital costs, especially for smaller or older plants [81].

### 4.2. Fuel Switching (Biomass, Hydrogen, Electrification)

As part of efforts to reduce emissions generated from fuel combustion for thermal energy generation, the cement industry is exploring various strategies. Transitioning from fossil fuels to alternative, low-carbon energy sources represent a primary strategy for mitigating CO_2_ emissions in cement production.

Utilising biomass as an alternative fuel in cement kilns has gathered considerable attention due to its potential to reduce net CO_2_ emissions, and in 2020, it formed 18% of the total thermal input across the U.K. cement sector [82]. Biomass sources, including agricultural residues, wood waste and energy crops, can be co-fired with conventional fossil fuels or used as the primary fuel source [83]. Biomass combustion is considered carbon-neutral since the CO_2_ released during the process is offset by the CO_2_ absorbed during biomass growth [84]. However, the U.K. needs more domestic biomass resources and the availability of sustainable biomass for the cement industry may be restricted by competing demands from other sectors [85,86].

Hydrogen emerges as another promising alternative fuel for cement production, offering the potential for near-zero emissions when produced from renewable sources or combined with CCS [87]. Its use in cement kilns shows promise for reducing CO_2_ emissions, as demonstrated by recent groundbreaking developments at Heidelberg Materials’ Ribblesdale plant in Lancashire. In 2021, hydrogen was utilised alongside other carbon-neutral fuels like meat and bone meal (MBM) and glycerine in a 100% net zero fuel mix to produce clinker. This innovative mix operated for three hours, with hydrogen contributing 39% of the heat, potentially leading to significant emissions reductions of up to 0.18 Mt CO_2_ annually [88]. Additionally, Cemex conducted earlier trials with hydrogen-enriched fuel mixes at its Alicante cement plant in Spain in 2019, successfully reducing CO_2_ emissions during cement production. By 2021, Cemex had deployed this technology across its European plants, including the Rugby cement plant in the U.K. [89,90].

However, hydrogen’s widespread adoption as a fuel in cement production relies on robust hydrogen infrastructure development and cost-effective production pathways [91,92].

Electrifying cement production processes, especially kiln systems, can contribute to decarbonisation by enabling the use of renewable or low-carbon electricity sources [93]. Nonetheless, the adoption of this solution could be improved by several factors. Firstly, the U.K. currently only generates 43% of electricity from renewable sources [94], indicating limited availability of renewable energy for industrial applications. Additionally, implementing electrification in the cement industry requires replacing current kilns with electric ones. However, this technology is still in its trial phase globally, with only one system worldwide capable of reaching the necessary temperatures while operating on renewable electricity [95,96]. These significant challenges could obstruct the implementation of electrification.

While electrification or alternative fuel adoption can eliminate emissions from the fuel source, they cannot mitigate process-related emissions inherent to raw materials. However, two principal approaches can reduce these emissions, including substituting clinker with alternative materials or employing CCUS [97].

### 4.3. Carbon Capture and Storage/Utilisation

CCS and CCU technologies have garnered significant attention as potential solutions for mitigating emissions from cement production, particularly those stemming from the calcination process.

Carbon capture involves capturing CO_2_ emissions from cement plants and transporting them to secure geological formations or depleted oil and gas reservoirs for long-term storage [97]. This approach holds promise for addressing both fuel combustion and calcination emissions, offering a pathway to deep decarbonisation for the cement industry. However, widespread CCS deployment faces challenges such as high capital costs, energy penalties and the availability of suitable storage sites [98].

Carbon utilisation involves capturing and converting CO_2_ emissions into valuable products or applications, such as construction materials, chemicals or fuels [99]. CCU presents economic incentives for deploying carbon capture technologies while contributing to circular economy initiatives. However, to achieve significant decarbonisation, CCU must complement CO_2_ storage, particularly in industries like cement production, where CO_2_ is an unavoidable by-product [38]. Therefore, a combination of CCU and permanent storage is necessary to address scalability and long-term viability challenges.

Regarding specific technologies, calcium looping with CaO-based materials emerges as the best method among others, as analysed by Antzaras et al. [100], balancing cost efficiency and environmental impact. While a technique like amine scrubbing would be initially more cost-effective, calcium looping’s lower operating costs (15% lower) and reduced environmental footprint (22% lower) make it a more sustainable and potentially economically viable option in the long term, especially with the anticipated rise in carbon taxes and the shift towards renewable energy sources.

The U.K. boasts a well-established CCUS policy framework and has invested significantly in CCUS research and demonstration projects [101]. However, deploying CCUS at a commercial scale in the cement industry remains challenging due to high costs and extensive infrastructure development requirements. Nonetheless, Heidelberg plans to establish the U.K.’s first carbon capture cement plant at Padeswood by 2027, aiming to capture 95% of CO_2_ emissions. Flue gases will circulate through an 80m high absorber column, bonding CO_2_ with amine. Captured CO_2_ will be compressed, purified and piped for storage beneath the Irish Sea, with the goal of capturing 800,000 tonnes annually at a cost of GBP 400 million [88].

### 4.4. Clinker Substitution

The clinker content per tonne of cement varies globally, ranging from 89% in the U.S. to 66% in China and approximately 77% in Europe. Despite a global average clinker factor of around 74%, it accounts for roughly 90% of cement’s carbon footprint [102]. Therefore, reducing the clinker content in cement is a well-established strategy for diminishing the carbon footprint of cement production. Globally, there is a discernible downward trend in the clinker-to-cement ratio owing to the increasing utilisation of blended cement and clinker substitutes. This shift is driven by the need to address long-term sustainability concerns, especially as industrial byproducts become less available. Substituting clinker with other cementation materials such as fly ash, GGBS and natural pozzolans emerges as a key strategy for reducing emissions in cement production [103,104]. These materials offer the potential to partially replace clinker in cement formulations while preserving or enhancing the final product’s performance characteristics.

In the U.K., major cement manufacturers have introduced low-carbon cement variants based on clinker and cement substitutions, summarised in Table 1, each with varying claims regarding emissions reduction. Among these variants, geopolymers stand out for their potential to reduce up to 90% of the carbon associated with traditional Portland cement [105]. Nevertheless, they are not readily available and only obtainable on a project-by-project basis, which is evaluated at the discretion of the manufacturer. Unlike other mixes in the standard, geopolymers almost eliminate the use of clinker and primarily utilise waste materials. While relatively cost-effective, the mass production of geopolymers requires expensive changes to current manufacturing plants due to the use of alkali-activated materials [106]. Additionally, there is a lack of standardisation under British Standards and CE marking certification. Addressing these gaps will require further research into their long-term performance, safety and compatibility with existing construction practices. Recent research has also explored cementless blended materials as complete replacements for traditional cement [107]. These materials have demonstrated the ability to achieve up to 90% of the compressive strength of conventional cementitious systems with the same water-to-cementitious ratio while omitting the use of alkali-activated materials, unlike geopolymers [108]. This development highlights a promising pathway for further reducing carbon emissions while maintaining structural performance.

Despite their environmental benefits, widespread adoption of clinker substitution strategies faces challenges. The availability of high-quality SCMs presents a significant barrier, as their production relies on other industrial processes such as coal-fired power plants and iron and steel production. Fluctuations in SCM production in the U.K. have been observed over the years, with the country’s relatively small iron and steel industry and declining number of coal-fired power plants impacting availability [6,109,110].

For instance, the U.K. slag production declined steadily in the past 15 years, dropping from 5 million tonnes in 2008 to just under 1.4 million in 2022. Similarly, figures for 2021 show U.K. fly ash production of 388,000 tonnes. These quantities are not remotely comparable to the U.K.’s 7 million tonnes of clinker produced annually. Yet, the U.K. Quality Ash Association estimates that approximately 100 million tonnes of fly ash are stockpiled in the U.K., presenting an opportunity for recovery and processing in cement and as secondary aggregates [16].

Conversely, limestone calcined clay cement or “LC3”, which consists of ground limestone and calcined clay, both available in abundance [111], emerged as a promising clinker substitute. It was used in the U.K. for the first time in 2023 [112], offering reduced embodied carbon by up to 40% and potential waste reduction benefits [6].

Moreover, efforts within the industry to explore new avenues for clinker and emissions reduction are ongoing. For instance, graphene is trialling graphene-enhanced cement at Breedon’s Hope plant in Derbyshire, offering a potential 10% strength increase and reducing material usage in structures [88].

**Table 1:** Green cement products [27,113,114,115,116,117].

Company	Product	Type	Impact Claim (vs. CEM I Concrete)
Aggregate Industries	ECOPlanet	CEM III *	30% less CO_2_
Breedon Cement	BREEDON Eco	CEM III	Lowers embodied CO_2_, no value
Cemex U.K.	Vertua	CEM III and geopolymer **	30% to 70% less embodied CO_2_
Heidelberg Materials	EcoCrete	CEM III and geopolymer	30% to 85% less emissions
Tarmac	CEVO	CEM II *** and geopolymer	Reduce emissions by up to 70%

* CEM III: Portland cement with 36–65% ground granulated blast furnace slag; ** CEM II: Portland cement with 12–20% limestone in combination with 12–20% of either ground granulated blast furnace slag, fly ash, natural pozzolana, natural calcined pozzolana or high reactivity natural calcined pozzolana; *** geopolymer: clinker-free alternative to cement. Geopolymers are the result of a reaction between solid aluminosilicate materials (such as fly ash, GBFS or naturally occurring metakaolin) and an alkaline solution (such as sodium silicate).

### 4.5. Other Innovations in Sustainable Cement Production

Various approaches beyond the methods above focus on innovative initiatives to transform cement production for sustainability. Highlighting noteworthy projects within the U.K., these advancements represent a significant stride towards reducing emissions and waste in the cement industry.

The Cement 2 Zero project leads a groundbreaking endeavour to produce carbon-neutral cement by recycling used cement and steel. Through the innovative Cambridge Electric Cement (CEC) process, this initiative could meet 25% of U.K. cement demand while offering a sustainable alternative to traditional cement, effectively minimising waste and emissions [118].

Likewise, Laing O’Rourke’s exploration of basalt fibre-reinforced polymer (BFRP) as a greener alternative for reinforcement in precast concrete showcases a significant breakthrough. Rigorous trials have demonstrated substantial reductions in embodied carbon, presenting potential carbon savings of up to 45% across the structural concrete unit [119].

Carbonation, a natural process in the lifecycle of concrete, involves the passive uptake of CO_2_ as it reacts with calcium hydroxide to form calcium carbonate. This phenomenon occurs gradually over the lifespan of concrete and has gained attention as a potential mechanism for offsetting emissions associated with cement production. While carbonation primarily impacts concrete as the final product, it plays a critical role in balancing the carbon budget of the cement and concrete value chain [120]. For example, studies have shown that significant quantities of CO_2_ can be reabsorbed over time by concrete structures, potentially contributing to net emissions reductions [121,122].

Building on this concept, active methods of leveraging carbonation have also emerged. For instance, the FastCarb project in France uses a reaction chamber where exhaust gases from cement kilns are passed over crushed recycled concrete [123]. This process enables accelerated carbonation, resulting in up to 50% CO_2_ capture within the concrete paste. Such approaches demonstrate how passive processes like carbonation can be harnessed and scaled through innovative engineering, offering promising opportunities for implementation in the U.K. cement industry and not merely as a “beyond net zero” measure, as considered in the MPA roadmap.

Similarly, the U.K. start-up Concrete 4 Change introduces CO_2_ mineralisation technology (mineral carbonation). This innovative approach accelerates the natural carbonation process, enabling concrete to act as an active CO_2_ sink while reducing overall emissions during production. Trials have shown that this technology can achieve a 20% reduction in carbon emissions, underscoring its potential as a dual-purpose solution for emission reduction and CO_2_ capture [124]. These products and initiatives demonstrate the potential of innovative technologies to drive sustainable practices in the U.K.’s cement sector.

Despite the U.K. cement industry’s strides in exploring and implementing decarbonisation technologies, significant challenges persist, ranging from technology readiness to resource availability and economic viability. Addressing these issues demands strategic planning and efforts from industry stakeholders, policymakers and researchers.

### 4.6. An Overview of the Cost vs. Impact of Cement Decarbonisation Options and Summary

**Figure 15:**
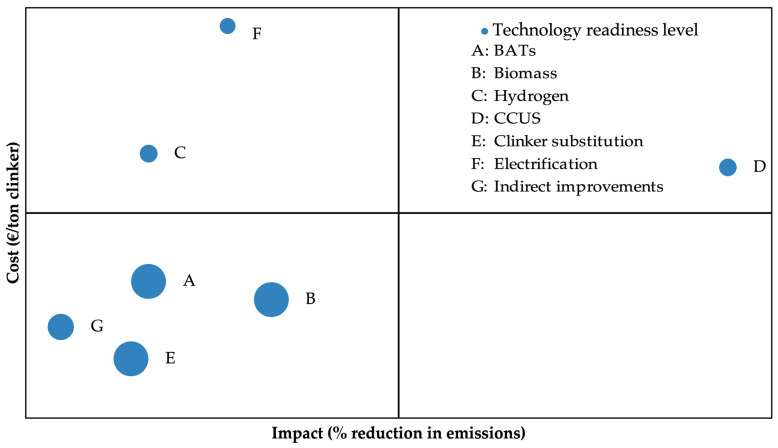
Cost vs. impact of the cement decarbonisation options where the bubble size indicates the TRL level. Data extracted from [93,125,126,127].

Figure 15 illustrates the correlation between the relative cost, average environmental impact and maturity level of the frequently cited decarbonisation options available for the cement industry.

This figure was created by compiling data from various sources on each option’s cost per unit of clinker, the percentage reduction in emissions and Technology Readiness Level (TRL) [93,125,126,127]. This approach allows for a comparison of options based on financial impact, environmental benefit and technology maturity, providing a snapshot of the current state of decarbonisation capabilities. It is important to note that such an assessment is inherently dynamic; what is shown here represents a snapshot of the current state of capabilities. As technologies advance and their TRLs increase, the positioning of these options within the quadrants may shift. New developments or improvements in existing technologies could lead to higher impacts or reduced costs over time, potentially changing the balance and availability of decarbonisation strategies.

Notably, the bottom-left quadrant encompasses options with low cost and low impact, where technologies such as clinker substitution (E), BATs (A), biomass (B) and indirect improvements (I) reside. Among these, clinker substitution offers a readily attainable option. At the same time, the use of biomass emerges as particularly promising due to its higher impact, relatively low cost and readiness for the technology. Moreover, establishing BATs will depend on the specific process, as the cost will vary depending on the changes needed in the plant. Indirect improvements, such as transportation and electricity, that involve mitigating emissions outside the scope of cement production are a viable consideration. Still, they are not necessary for the cement producers’ control.

The top-left quadrant comprises options with high cost and low impact, comprising electrification (F) and hydrogen (C). Electrification of cement production will be viable not only based on technical feasibility but also depending on the emissions associated with the electricity source. Similarly, using hydrogen as a fuel has less impact due to the trade-off occurring during its production, which requires substantial electricity. Additionally, they do not target the inherent process emissions of the production. Conversely, the top-right quadrant features options with high cost and high impact, with CCUS (D) representing a notable contender despite still being in the development stage. It has the highest impact and would directly handle the hard-to-utilise process emissions of cement production.

There are no options in the bottom-right quadrant representing low-cost, high-impact solutions, which makes sense given the nature of these technologies. Any options with such a favourable profile would likely be implemented as soon as they become available due to the significant benefit-to-cost ratio. This distribution underscores the complexity of decarbonisation efforts and emphasises the crucial role of strategic investments in advancing sustainable practices within the cement industry.

A summary of the reviewed technologies and options is presented in Table 2, assessing environmental effectiveness, technology readiness and implementation challenges based on the preceding discussion. Environmental effectiveness reflects the extent to which each technology reduces CO_2_ emissions, while technology readiness indicates the maturity of the solution. Implementation challenges assess factors such as cost, infrastructure requirements and supply limitations. A star rating system is employed to provide a comparative assessment across these three dimensions.

A five-star rating represents excellent performance, high readiness or minimal challenges, whereas a one-star rating denotes minimal contribution, early-stage development or severe implementation hurdles. For example, technologies with high capital or equipment costs may receive a lower score for implementation challenges, while solutions requiring extensive infrastructure upgrades face more significant adoption barriers and are rated accordingly. Conversely, products that align with existing production mechanisms but are still in the development stage are assigned a median score, reflecting the balance between the costs of experimenting, risks of early adoption and potential benefits. The ratings were assigned through a synthesis of qualitative and quantitative findings from the reviewed sources, ensuring consistency and objectivity.

BATs offer moderate environmental effectiveness and high readiness, improving energy efficiency and reducing emissions, but face high capital costs. Fuel switching, such as biomass and hydrogen, provides moderate to high environmental effectiveness and readiness but requires resource availability and infrastructure development. Carbon capture technologies have a high ecological impact but demand significant investment and infrastructure. Clinker substitution with SCMs and geopolymer cement shows substantial environmental benefits and readiness but faces supply and cost challenges. Innovative initiatives like cement recycling, CO_2_ mineralisation and graphene-enhanced cement are promising but need further development to overcome implementation barriers. These technologies represent a comprehensive approach to decarbonising the cement industry, balancing cost, impact and readiness to support a sustainable future. With consideration of the discussions presented in this section, strategies and recommendations for the U.K.’s cement sector are proposed in the next section.

## 5. Viable Strategies and Recommendations for Reduction of Cement Sector Emissions

The strategies presented in this section encompass a comprehensive approach towards mitigating emissions in the cement sector, covering immediate remedies, future considerations and policy implementations. In recognition of the urgent need to address environmental concerns, immediate actions are proposed to tackle current emissions sources and pave the way for sustainable practices. Furthermore, exploring future directions offers insights into innovative technologies and long-term strategies to achieve substantial emissions reductions over time. Additionally, governance and policy recommendations underscore the crucial role of regulatory frameworks and industry standards in driving systemic change and fostering a transition towards a greener cement sector. By examining strategies across these three dimensions, this section aims to provide a holistic understanding of the multifaceted efforts required to combat emissions effectively and steer the U.K.’s cement industry towards a more sustainable future.

### 5.1. Immediate Strategies

To achieve immediate reductions in emissions from the U.K. cement sector, the following two principal strategies emerge as practical and readily implementable within the existing industry framework.

#### 5.1.1. Increased Utilisation of Cementitious Materials

This strategy involves maximising the use of other cement constituents like fly ash and GGBS as partial clinker replacements in cement production. They can substitute up to 30–50% of clinker content [128], significantly decreasing the carbon footprint associated with the calcination process. Utilising the existing BS 8500-2:2019 [129] resourcefully, which includes a range of specified blended cements, offers a chance to boost environmental sustainability in the industry. Choosing cement with lower clinker content could reduce emissions without altering established standards [37]. There are various critical advantages to using this approach. Firstly, it reduces emissions, as lowering clinker content enables a proportional reduction in process-related CO_2_ emissions from calcination [29]. Secondly, embracing cementitious materials in concrete promotes circular economy principles, as these materials are byproducts from other industrial processes like coal combustion and steel production, thereby enhancing resource efficiency and waste valorisation. Thirdly, the utilisation of SCMs is supported by proven technology, with existing production facilities requiring relatively minimal modifications for implementation. Finally, from an economic standpoint, increasing SCM utilisation is economically feasible, as it necessitates minimal capital investment compared to alternative decarbonisation technologies while potentially reducing production costs through clinker substitution. This has been demonstrated in the Network Rail decarbonisation programme, achieving 51% carbon savings by utilising an 80% GGBS product [130].

#### 5.1.2. Optimisation of Clinker Manufacturing and Use in Concrete Production

This approach encompasses optimising the manufacturing process of clinker and its utilisation in concrete production. Firstly, it aims to enhance the efficiency of clinker manufacturing by optimising thermal energy efficiency, process control and productivity. Variable coal feeding rate (VCFR) control can improve energy distribution while focusing on the raw material preheating and decomposition process unit yields significant benefits [131,132]. Advanced process control (APC), like Model Predictive Control, reduces energy consumption and maintains quality standards [133]. Secondly, the efficiency of clinker utilisation in concrete manufacturing could be maximised through techniques like particle packing optimisation, the use of dispersants and the incorporation of fillers [29]. By reducing the clinker factor (clinker/cement ratio), the carbon intensity of concrete can be lowered without compromising performance.

These options represent practical steps that can be implemented incrementally within the cement industry’s existing framework. As such, they present realistic pathways toward achieving meaningful emissions reductions in the near to medium term, laying the foundation for further innovation and sustainability improvements in the future.

#### 5.1.3. Biomass as Fuel

Research indicates that biomass can effectively replace a significant portion of fossil fuels in cement production, reducing emissions and lowering production costs [83]. Various types of biomass, including food residue biomass [134] and non-wood biomass combustion ash [135], have been identified as viable alternative fuels in the cement industry. Moreover, using wood waste ash as a partial cement replacement material in producing structural-grade concrete has shown promising results in strength and durability [136].

Recognising the competing demands from other sectors for biomass in the U.K. is significant. Therefore, it is essential to consider sustainable sourcing and management of biomass, as highlighted by recent studies [137], with some suggesting the need for bioenergy sustainability schemes [138]. Biomass as fuel should be integrated with other renewable energy forms to create a robust and diverse energy strategy.

### 5.2. Near-Term Considerations and Future Recommendations

As the U.K. cement industry continues its decarbonisation journey, several considerations and recommendations emerge for the near and long term.

#### 5.2.1. Exploration of Alternative SCM Sources

The revised BS 8500 in 2023 introduces “ternary cement combinations” with Portland cement and two additional binder materials, potentially altering the role of traditional CEM I [139,140]. While historically ensuring concrete safety, BS 8500’s limitations sometimes hindered innovation, especially in carbon reduction. The latest version permits up to two additional constituents, focusing on including limestone. This opens potential avenues for new products utilising currently available SCMs. Therefore, actively exploring alternative sources becomes imperative given the limited availability of high-quality SCMs like GGBS and fly ash within the U.K. Potential avenues include the following:

- Recovery and processing of stockpiled fly ash from legacy coal combustion activities;
- Utilisation of calcined clays and other natural pozzolanic materials abundant in the U.K.;
- Collaboration with other industries to identify and valorise suitable by-product streams.

Diversifying the supply of clinker substitutes will support long-term decarbonisation efforts and contribute to resource efficiency and waste minimisation.

#### 5.2.2. Adoption of Innovative Cement Formulations

Although SCM incorporation offers practical solutions, the development and widespread adoption of novel, low-carbon cement formulations like LC3 and geopolymers should be actively pursued [141]. The alternative binders such as microbial cements (MCs), calcium aluminates cements (CACs), geopolymer cements and super sulphated slag cements (SSCs) have the potential to significantly reduce the carbon intensity of cement production, offering up to 90% emissions reduction compared to traditional Portland cement [142,143].

#### 5.2.3. BATs Implementation

Adopting BATs across cement manufacturing plants presents a significant opportunity to drive energy efficiency improvements, reduce emissions and optimise resource utilisation in the near to medium term. BATs encompass diverse technological solutions and operational practices tailored to specific processes and plant configurations [38].

In the U.K. context, major cement manufacturers like CEMEX and Heidelberg Materials have made notable investments in upgrading equipment and implementing BATs within their facilities [79,80]. However, the widespread adoption of cutting-edge technologies across the nation’s cement plants faces challenges, particularly concerning high capital costs, especially for smaller or older plants. Overcoming these barriers will require supportive policy measures, such as financial incentives and regulatory frameworks that encourage the adoption of BATs throughout the industry [78].

By leveraging the full potential of BATs, the U.K. cement industry can substantially improve energy efficiency, reduce emissions and optimise resources, paving the way for a more sustainable and environmentally responsible sector. However, concerted efforts from industry stakeholders, policymakers and researchers will be necessary to address the challenges and facilitate the widespread implementation of these best practices across the nation’s cement manufacturing facilities.

#### 5.2.4. Development of CCUS Infrastructure

While alternative cement formulations offer long-term decarbonisation potential, CCUS technologies remain an essential interim solution for mitigating emissions from existing cement production processes. The U.K. cement industry should collaborate with government agencies and other stakeholders to develop the necessary infrastructure for large-scale CCUS deployment [144,145]. Key considerations include the following:

- Establishing pipelines and identifying suitable geological storage sites or utilisation pathways for CO_2_ transportation and storage infrastructure;
- Continue research and development efforts to drive down the capital and operational costs associated with CCUS implementation with a focus on calcium looping technology;
- Implementing supportive policies, such as carbon pricing mechanisms and incentives, to enhance the economic viability of CCUS projects.

By addressing these considerations, the U.K. can position itself as a leader in CCUS deployment, enabling the cement industry to achieve significant emissions reductions while transitioning to more sustainable production methods.

Tackling these matters requires collaborative efforts across the stakeholder network, including collaboration between industry players, government bodies, research institutions and financial entities. Joint initiatives aimed at knowledge sharing, technology transfer and funding support are imperative for accelerating the transition to sustainable cement production.

### 5.3. Demand-Driven Innovations and Policy Solutions

While technological advancements and production process improvements are crucial, demand-side interventions and policy measures will be pivotal in accelerating the transition to a low-carbon cement industry in the U.K.

#### 5.3.1. Fostering Collaboration and Innovation

Achieving deep decarbonisation in the cement industry requires collaborative efforts across the entire stakeholder network, from raw material suppliers to end-users:

- Public–private partnerships: Facilitating collaboration between industry, academia and government agencies can accelerate research, development and knowledge transfer at a large scale;
- Innovation incentives: Providing financial incentives, such as grants, tax credits or low-interest loans, can encourage industry players to invest in decarbonisation technologies and innovative processes;
- Knowledge-sharing platforms: Establishing platforms for sharing best practices, case studies and lessons learned can promote cross-pollination of ideas and accelerate the adoption of successful strategies.

By fostering an ecosystem of collaboration and innovation, the U.K. can harness various stakeholders’ collective expertise and resources, driving transformative change within the cement industry.

#### 5.3.2. Supportive Regulatory and Policy Framework

A robust regulatory and policy framework is essential to create an enabling environment for the cement industry’s decarbonisation efforts:

- Regulatory standards and guidelines: Updating building codes, material standards and policies to incorporate sustainability considerations can drive demand for low-carbon cement and concrete products [146]. Specifically, the inclusion of alternative binders such as geopolymers in material standards like BS EN 197 and CE marking frameworks will be vital for ensuring their acceptance and widespread use in the construction sector;
- Targeted financial incentives: Offering tax incentives, subsidies or other financial support mechanisms can offset the initial costs of adopting decarbonisation technologies and processes;
- Research and development funding: Allocating dedicated funding for research into novel cement formulations, alternative raw materials and innovative production methods can foster technological breakthroughs.

By creating a supportive regulatory and policy landscape, the U.K. government can provide the necessary momentum for the cement industry to embrace sustainable practices and accelerate the transition towards a low-carbon future.

#### 5.3.3. Upstream and Downstream Decarbonisation Strategies

Addressing carbon emissions in the cement industry requires a comprehensive approach encompassing upstream and downstream strategies. By focusing on the supply chain and the end-use applications of cement, the industry can achieve more substantial and lasting reductions in its overall carbon footprint [147].

- Upstream

Decarbonising the supply chain is a critical step in reducing the overall carbon footprint of the cement industry. This involves targeting emissions in transportation, electricity production and material sourcing [127,148,149]. Specifically, it includes enhancing the efficiency of transportation networks to reduce fuel consumption and emissions, transitioning to renewable energy sources for electricity used in material processing and sourcing materials from suppliers who practice sustainable methods. These efforts represent upstream decarbonisation strategies, focusing on reducing emissions in the supply chain leading up to cement production.

- Downstream

Moreover, since cement is a key component of concrete, it is crucial to implement downstream decarbonisation strategies for the concrete sector as part of broader efforts to mitigate emissions associated with cement production. Several strategies can significantly contribute to lowering the carbon footprint of concrete, thereby indirectly benefiting the cement industry. These strategies include optimising concrete mix design to minimise cement usage, reducing over-specification in construction projects and improving the design of structural elements to use materials more efficiently. Extending the lifespan of buildings also reduces the need for new cement production over time.

Additionally, carbonation, the natural process by which atmospheric CO_2_ reacts with concrete over its lifecycle, can contribute to offsetting a portion of emissions associated with cement production. While carbonation primarily functions as a passive and long-term process, recent innovations, such as accelerated carbonation techniques, show potential for enhancing its impact [122,123].

Furthermore, exploring alternative construction materials and leveraging the thermal mass properties of concrete can further reduce overall emissions. By integrating these concrete-focused strategies, the cement sector can achieve more comprehensive and effective decarbonisation outcomes [125,150,151].

### 5.4. Roadmap to Net Zero

**Figure 16:**
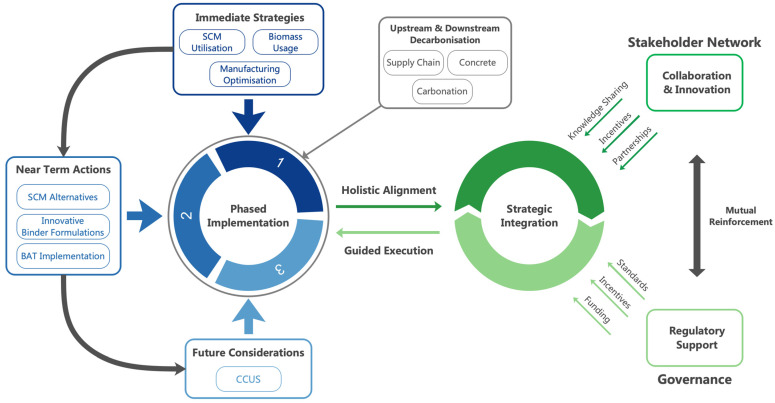
A phased roadmap for achieving net zero emissions in the U.K. cement industry, outlining immediate actions, near-term strategies and long-term measures. The roadmap emphasises technological innovation, stakeholder collaboration and regulatory support to drive holistic and strategic decarbonisation.

Achieving net zero emissions by 2050 for the U.K. cement industry demands a comprehensive and phased roadmap that integrates immediate actions, near-term strategies and long-term transformative measures, as discussed in the preceding sections. This roadmap, illustrated in Figure 16, was created by analysing key decarbonisation pathways for the sector, drawing on emerging research, industry reports and input from technological frameworks. It prioritises solutions based on their maturity, feasibility and potential impact, mapping out actions across different time horizons. This multi-pronged approach leverages technological innovations, operational optimisations and supportive policies to drive decarbonisation across the sector.

The roadmap commences with Phase 1, which emphasises implementing readily available and economically viable solutions to reduce emissions while establishing a solid foundation for future advancements. During this initial phase, key strategies include maximising the utilisation of readily available SCMs to lower clinker content, optimising clinker manufacturing processes through enhanced efficiencies and productivity gains and increasing the use of alternative fuels such as biomass and waste-derived inputs.

Phase 2 focuses on scaling up transformative low-carbon technologies and driving widespread commercialisation. This phase involves adopting BATs and operational best practices, exploring existing underutilised reserves of SCMs like stockpiled fly ash and natural pozzolanic materials and large-scale adoption of innovative cement formulations like LC3 and geopolymers. Fostering public–private partnerships, providing innovation incentives and establishing knowledge-sharing platforms will drive technological advancements and knowledge dissemination during this phase.

The final Phase 3 aims to achieve net zero emissions by 2050 and potentially progress towards harmful emissions through continuous innovation and optimisation. Key strategies include maximising the deployment of CCUS technologies across all cement plants, coupled with the utilisation of captured CO_2_ in value-added applications and establishing a supportive regulatory and policy framework that incentivises sustainable practices, promotes research and development and aligns with the U.K.’s broader net zero ambitions.

Implementing these phases will align with efforts to decarbonise the upstream and downstream strategies, focusing on the supply chain and the end-use applications of cement. Although cement producers may not directly control these elements, they can influence them by prioritising suppliers committed to sustainable practices. Cement producers can drive broader industry-wide improvements by limiting partnerships with less sustainable stakeholders.

Navigating this transformative journey towards a decarbonised cement industry in the U.K. will require concerted efforts from all stakeholders, including cement manufacturers, policymakers, research institutions and end-users. Collaboration, knowledge sharing and a collective commitment to sustainability will be paramount in overcoming challenges and seizing opportunities that emerge along the way. By employing this comprehensive roadmap, the U.K. cement industry can position itself as a global leader in sustainable cement production, aligning with the nation’s climate change goals and contributing to a more sustainable future.

## 6. Conclusions

Decarbonising cement production presents a formidable challenge that requires urgent, multi-dimensional and transformative action. This review has evaluated key decarbonisation technologies, strategies and their implications within the U.K. cement industry, identifying pathways to align the sector with net zero emission targets.

The findings highlight that while initiatives focused on enhancing energy efficiency have yielded improvements, addressing process emissions from calcium oxide production has emerged as a critical priority. Strategies that target clinker content reduction, such as increasing the utilisation of SCMs and developing low-carbon cement formulations (e.g., LC3 and geopolymers), offer significant emissions reduction potential but require advancements in resource availability, product standards and economic feasibility to achieve widespread adoption. The role of CCUS stands out as a promising yet demanding solution, capable of mitigating both combustion and calcination emissions. However, its deployment at scale demands robust infrastructure, financial support and clear regulatory frameworks. Similarly, alternative fuels such as biomass and hydrogen, alongside the electrification of processes powered by renewable energy, hold promise but face challenges, including supply limitations, infrastructure gaps and high resource demands.

The broader implications of these findings suggest that achieving decarbonisation goals will require a phased and integrated approach. Immediate actions, such as optimising clinker processes and increasing SCM use, must be complemented by long-term investments in innovative cement formulations, CCUS and infrastructure upgrades. Crucially, fostering a supportive ecosystem through stakeholder collaboration, innovation incentives, and regulatory alignment is essential to overcoming technological and economic barriers.

This review contributes to the field by offering a comprehensive analysis of decarbonisation pathways specific to the U.K. cement sector. It underscores the need for policy-driven transformation and highlights opportunities for technological and operational advancements. By addressing both immediate challenges and long-term opportunities, the U.K. cement industry can serve as a model for other hard-to-abate sectors, demonstrating that a sustainable, low-carbon future is achievable through collective and sustained efforts.

## Figures and Tables

**Table 2:** Overview of decarbonisation technologies in the cement industry (star rating indicates relative performance in environmental effectiveness, technology readiness and implementation challenges. ★ indicates a higher rating and ☆ indicates a lower rating).

	Technology	Environmental Effectiveness	Technology Readiness	Implementation Challenges	Comments
BATs	Clinkerisation	★★★☆☆	★★★★★	★★★☆☆	High capital costs
	Grinding	★☆☆☆☆	★★★★★	★★★☆☆	High capital costs
	Generic	★★☆☆☆	★★★★★	★★☆☆☆	Capital costs
Fuel	Biomass	★★★☆☆	★★★★☆	★★☆☆☆	Supply limitations
	Hydrogen	★★★★☆	★★★☆☆	★★★★☆	Infrastructure, cost
	Electrification	★★★★☆	★★☆☆☆	★★★★☆	Maturity, energy supply
CC	Calcium looping	★★★★★	★★★☆☆	★★★★☆	High capital costs
Clinker	Geopolymer cement	★★★★☆	★★★★☆	★★★★☆	Plant modification costs
	SCMs	★★★☆☆	★★★★★	★★☆☆☆	Supply, quality
	Geopolymer cement	★★★★★	★★★☆☆	★★★☆☆	Supply, quality
	LC3	★★★★☆	★★★★☆	★★☆☆☆	Early adoption phase
Initiatives	Cement recycling	★★★☆☆	★★☆☆☆	★★★☆☆	Development phase
	CO_2_ mineralisation	★★★★☆	★★★☆☆	★★☆☆☆	Early adoption phase
	Graphene-enhanced cement	★☆☆☆☆	★★☆☆☆	★★★☆☆	Development phase

## Data Availability

All data supporting this study are provided in full in this paper.

## List of Abbreviations

APC	Advanced process control	LC3	Limestone calcined clay cement
BATs	Best available techniques	LCA	Life cycle analysis
BFRP	Basalt fibre-reinforced polymer	MCs	Microbial cements
CaCO_3_	Calcium carbonate	MBM	Meat and bone meal
CACs	Calcium aluminates cements	MPA	Mineral Products Association
CaO	Calcium oxide	SCM	Supplementary cementitious material
CCUS	Carbon capture utilisation and storage	SCR	Selective catalytic reduction
CEC	Cambridge Electric Cement	SSCs	Super sulphated slag cements
GCCA	Global Cement and Concrete Association	TRL	Technology Readiness Level
GGBS	Ground granulated blast furnace slag	VCFR	Variable coal feeding rate
GHG	Greenhouse gases	U.K.	United Kingdom
